# The effect of hypoxic interventions on swimming performance in competitive athletes: a systematic review and meta-analysis

**DOI:** 10.3389/fphys.2026.1755641

**Published:** 2026-02-09

**Authors:** Jiangzhou Chen, Taofeng Xing, Jianan Gong, Ditao Song

**Affiliations:** 1 Department of Health and Exercise Science, Graduate School, Inha University, Incheon, Republic of Korea; 2 Department of Sports & Leisure, College of Humanities, Daegu University, Gyeongsan, Republic of Korea; 3 Department of Sports Science, Graduate School of Chonbuk National University, Jeonju, Republic of Korea; 4 Department of Physical Education and Health Sciences, Guangxi University of Science and Technology for Nationalities, Laibin, China

**Keywords:** competitive athletes, hypoxic interventions, physiological outcomes, simulated altitude, swimming performance

## Abstract

**Objectives:**

To evaluate the effects of hypoxic interventions (HI) on swimming performance and physiological outcomes in competitive swimmers.

**Design:**

Systematic review and meta-analysis following PRISMA guidelines and registered in PROSPERO (CRD420251170303).

**Methods:**

Seven databases were searched to identify controlled trials comparing hypoxic and normoxic training under identical conditions. Eleven studies (n = 182 swimmers) were included. Standardized mean differences (SMD) and 95% confidence intervals (CI) were calculated using a random-effects model. Subgroup analyses were performed by event distance and simulated altitude.

**Results:**

HI produced a small but significant improvement in swimming performance (SMD = −0.34, 95% CI [−0.62, −0.06], p = 0.02) with low heterogeneity (I^2^ = 30%). No significant changes were observed for VO_2_max, HRmax, or VEmax (all p > 0.05). Subgroup analyses indicated greater benefits for 100 m and 200 m freestyle and for interventions conducted at simulated altitudes ≥3500 m.

**Conclusion:**

Hypoxic interventions yield meaningful yet modest enhancements in competitive swimming performance, likely through non-hematological adaptations such as improved muscle oxygen utilization and fatigue tolerance. Tailoring HI protocols to event-specific demands and sufficient hypoxic stimulus levels may optimize outcomes.

**Systematic Review Registration:**

Registered on PROSPERO (registration number: CRD420251170303).

## 1 Introduction

Swimming performance at the competitive level emerges from the interaction of technical proficiency, neuromuscular power, and aerobic–anaerobic energy supply (Ruiz-Navarro et al., 2025). Among the many strategies proposed to enhance these determinants, hypoxic interventions (HI)—broadly defined as training or exposure performed under reduced inspired oxygen—have attracted sustained interest from coaches and sport scientists (Czuba et al., 2017). Classic paradigms include “live high–train high” (LHTH) and “live high–train low” (LHTL), whereas more recent “live low–train high” (LLTH) variants implement hypoxia acutely during selected sessions (e.g., intermittent hypoxic training, high-intensity interval work in hypoxia, or repeated-sprint training in hypoxia) (Behrendt et al., 2022). These approaches aim to amplify training stimuli through altered oxygen availability, with putative benefits spanning hematological, cardiorespiratory, peripheral muscular, and perceptual domains (Millet and Brocherie, 2020).

For swimmers, the rationale for HI is compelling but nuanced (Papadimitriou et al., 2023). Unlike many land-based sports, competitive swimming occurs in a medium that constrains breathing patterns and elevates the cost of propulsion, while race outcomes hinge on tight margins across events from 50 m sprints to 400 m middle distances (Liu et al., 2025). Hypoxia could plausibly improve sea-level performance via multiple pathways: modest hematological or ventilatory adaptations, improved muscle oxygen utilization and buffering capacity, altered pacing strategies, and/or enhanced tolerance to repeated high-intensity efforts with restricted recovery (Brocherie et al., 2017). At the same time, inconsistent findings in the literature suggest that benefits may depend on the congruence between the hypoxic stimulus (e.g., simulated altitude, dose, and work-to-rest structure) and the event-specific demands of swimming, as well as on methodological quality and reporting standards across trials (Millet et al., 2010; Wilber et al., 2007).

Several systematic reviews and meta-analyses have examined hypoxic or altitude-related training strategies in endurance and team-sport athletes (Hamlin et al., 2017; Bonetti and Hopkins, 2009); however, competitive swimming has often been underrepresented or pooled with other sport modalities (Álvarez-Herms et al., 2019; Feng et al., 2023). Given the substantial differences in technical structure, energetic demands, and breathing constraints between swimming and land-based sports, the applicability of conclusions drawn from such mixed-sport syntheses to swimming-specific practice remains limited (Born et al., 2024). In particular, previous reviews have seldom focused on swimming race performance itself, nor systematically explored whether the effects of HI differ across swimming event distances.

Evidence specific to swimmers has historically been fragmented across paradigms—ranging from continuous or interval training performed in hypoxia, to intermittent hypoxic exposure adjuncts, to repeated-sprint protocols delivered under low FiO_2_ (Czuba et al., 2017; Camacho-Cardeñosa et al., 2020; Goliniewski et al., 2024). Studies vary in simulated altitude (≈2,300–5,500 m), mesocycle length (≈3–8 weeks), weekly frequency, session duration, and outcome selection (e.g., 50/100/200/400 m freestyle times, VO_2_max, HRmax, VEmax). Such diversity complicates synthesis and can obscure practical guidance for coaches who must allocate scarce training time within periodized plans leading into competition (Goliniewski et al., 2024; Joyce et al., 2025). Importantly, performance improvements following HI are not consistently accompanied by parallel changes in conventional cardiopulmonary markers, suggesting that the inclusion of multiple physiological outcomes alongside race performance may help clarify whether observed effects are driven primarily by central cardiovascular adaptations or by peripheral and neuromuscular mechanisms (Yi et al., 2024; Uchimaru et al., 2023).

Furthermore, prior work in endurance and team-sport settings indicates that HI effects on performance are not always paralleled by consistent changes in conventional physiological markers (e.g., VO_2_max), raising the possibility that non-hematological adaptations—economy, neuromuscular efficiency, buffering/tolerance, or race-execution factors—may mediate performance gains (Nugent et al., 2017). For swimming in particular, where breathing mechanics and hydrodynamic constraints differ from running or cycling, the transferability of land-sport evidence is uncertain (Ohya et al., 2021). In addition, the responsiveness to HI may vary across swimming events. The 50 m freestyle is dominated by start, underwater phases, and explosive propulsion with a predominant reliance on phosphagen and anaerobic energy supply, whereas the 100 m and 200 m freestyle involve a greater interaction between anaerobic and aerobic metabolism and repeated high-intensity efforts under incomplete recovery (Hellard et al., 2018; Peyrebrune et al., 2014). The 400 m freestyle further increases aerobic contribution while maintaining substantial demands on fatigue tolerance (Campos et al., 2017; Swanwick and Matthews, 2018). Examining multiple race distances therefore allows assessment of whether HI preferentially benefits speed-endurance–oriented events rather than pure sprint performance. A focused, swimmer-specific synthesis is therefore warranted to clarify whether HI confers a measurable performance advantage at sea level, and under what conditions.

The present study systematically reviews and meta-analyzes randomized and non-randomized controlled trials in competitive swimmers that compare hypoxic versus normoxic conditions, while holding other training and living variables constant. We evaluate effects on primary outcomes of swimming performance and secondary physiological outcomes (VO_2_max, HRmax, VEmax). By restricting the analysis to competitive swimmers and applying distance-specific and altitude-based subgroup analyses, this study advances previous meta-analyses from a generalized evaluation of hypoxic training to a competition-relevant, event-specific synthesis. Given the event-specific technical and energetic profiles in swimming, we also examine whether effects differ by race distance (50/100/200/400 m freestyle) and by the magnitude of the hypoxic stimulus (simulated altitude).

## 2 Methods

This study followed the Preferred Reporting Items for Systematic Reviews and Meta-Analyses (PRISMA) guidelines (Page et al., 2021) and was registered in PROSPERO (Record URL: https://www.crd.york.ac.uk/PROSPERO/view/CRD420251170303).

### 2.1 Eligibility criteria

Studies will be included if they meet the following criteria: (1) participants are competitive swimmers who are healthy, have intact limbs, and no history of disease; (2) the study design is a parallel-group trial, either randomized or non-randomized; (3) the intervention group undergoes training under hypoxic conditions, such as interval training, repeated sprint training, individualized training, conventional training, or hypoxic exposure, while the control group differs only in oxygen concentration during training or exposure, with all other training and living conditions remaining identical; (4) the primary outcome includes at least one swimming performance measure, and secondary outcomes may include stroke rate, stroke length, maximal oxygen uptake (VO_2_max), maximal heart rate (HRmax), and maximal ventilation (VEmax), with sufficient data provided to calculate effect sizes; (5) the full text or the necessary data for this review is available.

Studies will be excluded if they meet the following conditions: (1) they do not meet the inclusion criteria; (2) HI are primarily based on breath-holding that induces acute hypoxia rather than physiological adaptive hypoxia; (3) primary outcomes lack the data required to calculate effect sizes.

### 2.2 Information sources

An independent researcher conducted Boolean searches across seven databases: Web of Science, PubMed (including MEDLINE and PubMed Central), SPORTDiscus, ScienceDirect, Cochrane Library, Embase, and ProQuest. No restrictions were imposed on language, publication type, or publication date. The scope of the retrieved literature is from the establishment of the database to September 5, 2025. In addition, supplementary resources such as Google Scholar and ResearchGate were consulted, and the reference lists of relevant articles were manually screened to identify additional eligible studies.

### 2.3 Search strategy

The search string was: (IHT OR “intermittent hypoxic training” OR “intermittent hypoxia training” OR HIIT OR “high-intensity interval” OR “high intensity interval” OR “sprint interval” OR sprint* OR “repeated sprint” OR RSH OR “exercise training”) AND (swim* OR swimmer* OR “swimming performance” OR breaststroke OR butterfly OR freestyle OR backstroke). Database-specific search settings were applied, and the detailed search strategies are provided in Supplementary Appendix B. Two independent researchers imported the retrieved records into EndNote X9 for reference management and duplicate removal. Subsequently, titles and abstracts were independently screened according to the predefined inclusion criteria. All reviewers received standardized training prior to screening to ensure consistency. In cases of disagreement between the two reviewers, a third researcher was consulted to resolve discrepancies and reach consensus. After the initial screening, full texts of potentially eligible studies were assessed for eligibility.

### 2.4 Risk of bias

This study used the Cochrane Risk of Bias Assessment Tool version 2.0 (RoB 2) (Higgins et al., 2025) to evaluate the risk of bias in the included randomized controlled trials and clinical controlled trials, and the robvis tool was employed to generate visualizations (McGuinness and Higgins, 2020). This domain-based tool assesses five specific sources of bias: (1) bias arising from the randomization process; (2) bias due to deviations from intended interventions; (3) bias resulting from missing outcome data; (4) bias in the measurement of outcomes; and (5) bias in the selection of the reported results. Each domain includes a series of signaling questions. After completing the assessment of these questions, the risk of bias is judged and assigned to one of three levels: “low risk of bias,” “some concerns,” or “high risk of bias” (Higgins et al., 2025). The assessment was conducted independently by two reviewers, and any disagreements were resolved through discussion with a third reviewer until consensus was reached.

### 2.5 Data extraction process

Each study was coded according to the following variables: author, participant characteristics (Subject, sex, age, training experience), Experimental/Control Group Treatment, Hypoxia Parameters, training frequency, session duration, and outcomes (Table 1). Data were independently extracted by two reviewers. Discrepancies were resolved through discussion, and when necessary, arbitration by a third reviewer. Although inter-rater reliability statistics were not calculated, all disagreements were resolved prior to data synthesis to ensure consistency and objectivity. If baseline values of outcome measures showed significant between-group differences, the corresponding results were excluded from the analysis. In studies with three or more groups (e.g., two experimental groups and one control group), only the groups serving as comparators to the HI were included, whereas groups with incomplete data or protocol deviations (e.g., missing follow-up data) were excluded from the analysis. When studies presented swimming performance using bar charts with error bars but did not report exact means and standard deviations, two researchers extracted the data using the WebPlotDigitizer tool (Li et al., 2025).

**TABLE 1:** Basic characteristics of included studies in the meta-analysis (n = 11). M mean, SD standard deviation, FTT freestyle test time.

Study	Subject (Sex)	Age (M ± SD)	Experimental/Control group treatment	Hypoxia parameters	Training frequency	Session duration	Outcomes
Rodríguez et al. (2015)	27 (9 m)	G1: m = 21.2 ± 2.5 f = 20.2 ± 2.6 G2: m = 19.7 ± 2.7 f = 19.6 ± 3.1	Hypoxic personalized training/normoxic personalized training	Highland 2320 m	18 times per week of 4 weeks	Not reported	50 m FTT 100 m FTT 200 m FTT 400 m FTT
Park and Lim (2017)	20 (10 m)	G1: 22.5 ± 2.6 G2 22.9 ± 3.9	Hypoxic personalized training/normoxic personalized training	Simulated 3000 m	3 times per week of 6 weeks	120 min	50 m FTT 400 m FTT VO_2_max
Park et al. (2018a)	20 (10 m)	G1: 22.5 ± 2.6 G2: 22.9 ± 3.9	Hypoxic personalized training/normoxic personalized training	Simulated 3000 m	3 times per week of 6 weeks	90 min	400 m FTT VO_2_max
Park et al. (2018b)	20 (10 m)	G1: 22.5 ± 2.6 G2: 22.9 ± 3.9	Hypoxic personalized training/normoxic personalized training	Simulated 3000 m	3 times per week of 6 weeks	90 min	400 m FTT VO_2_max
Truijens et al. (2003)	16 (6 m)	G1: 28.8 G2: 28.9	Hypoxic high-intensity training + normoxic regular training/normoxic high-intensity training + normoxic regular training	Simulated 2500 m	3 times per week of 5 weeks	Not reported	100 m FTT 400 m FTT HRmax VEmax
Miller and George (2012)	8 (8 m)	20 ± 2	Hypoxic exposure + regular aerobic training/regular aerobic training	Simulated 2300 m	3 times per week of 3 weeks	60–120 min	100 m FTT
Czuba et al. (2017)	15 (15 m)	G1: 19.1 ± 1.3 G2: 20.5 ± 1.3	High-intensity intermittent hypoxia training/high-intensity intermittent normoxia training	Simulated 2500 m	2 times per week of 4 weeks	65–75 min	100 m FTT 200 m FTT VO_2_max HRmax VEmax
Rodríguez et al. (2007)	12 (7 m)	20.2 ± 8.8	Intermittent hypobaric hypoxia exposure + normoxic regular training/normoxia exposure + normoxic regular training	Simulated 4000–5500 m	5 times per week of 4 weeks	180 min	100 m FTT 400 m FTT VO_2_max HRmax VEmax
Kim et al. (2021)	20 (20 m)	G1: 24.00 ± 3.06 G2: 23.90 ± 3.07	Hypoxic personalized training/normoxic personalized training	Simulated 3000 m	3 times per week of 6 weeks	120 min	400 m FTT VO_2_max
Pupišová et al. (2024)	8 (5 m)	15.75	Intermittent hypoxia training/normoxic regular training	Simulated 3500–4000 m	6 times per week of 3 weeks	60 min	50 m FTT
Amir et al. (2016)	16 (0 m)	G1: 14.4 ± 1.07 G2: 14 ± 1.05	Hypoxic repeated sprint training/normoxic repeated sprint training	Simulated 3500 m	5 times per week of 8 weeks	Not reported	100 m FTT 200 m FTT

### 2.6 Synthesis of results

Review Manager (RevMan) version 5.4 was used to perform the overall effect analysis of the extracted data and to generate forest plots and funnel plots. Sensitivity analyses, Egger’s test, and subgroup analyses were conducted using Stata version 17.0. Given the considerable heterogeneity among the HI protocols, a random-effects model was applied for the analysis. Heterogeneity was assessed using the chi-square test (p value) and the I^2^ statistic, with p > 0.1 and I^2^ < 40% considered indicative of low heterogeneity (Deeks et al., 2025). Because the included studies involved different swimming events, standardized mean differences (SMD) were used for pooled analysis. SMD values were classified as trivial (<0.2), small (0.2–0.49), moderate (0.5–0.79), and large (≥0.8) (Cohen, 1988). A p value <0.05 was considered statistically significant.

In addition, subgroup analyses were conducted based on two potential moderators—swimming event and simulated altitude—to examine their respective effects. For the swimming event subgroup, mean differences (MD) were used for pooled effect sizes because the evaluation criteria were consistent within the same event, whereas SMD were applied in the other subgroups.

## 3 Results

### 3.1 Study characteristics

**FIGURE 1:**
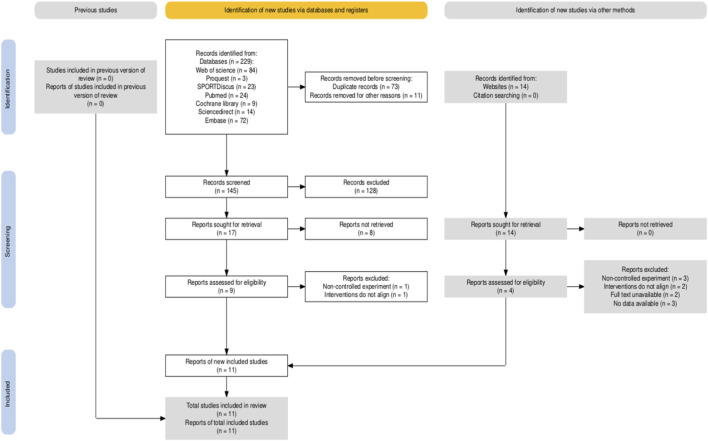
Article retrieval and filtering flow.

The flow diagram illustrates the entire process of the systematic search (Figure 1). Database searches initially identified 229 potentially relevant articles. After successive screening steps, seven studies (Hellard et al., 2018; Peyrebrune et al., 2014; Campos et al., 2017; Swanwick and Matthews, 2018; Page et al., 2021; Higgins et al., 2025; McGuinness and Higgins, 2020) met the inclusion criteria, and four additional studies (Li et al., 2025; Deeks et al., 2025; Cohen, 1988; Rodríguez et al., 2015) were identified through supplementary sources. In total, 11 studies were included (Table 1). The included studies comprised 182 participants, with 94 in the experimental groups and 88 in the control groups. The basic characteristics of the studies are as follows: with respect to sex, seven studies included both male and female participants, three studies reported males only, and one study reported females only. Regarding age, nine studies were classified as involving adults and two as involving minors. All 182 participants were competitive swimmers with established swimming experience. In terms of training frequency, all 11 studies implemented training lasting 3–8 weeks, with 3–18 sessions per week. For session duration, two studies did not report the length of individual sessions, while the remaining nine studies reported durations of at least 60 min. With respect to outcomes, all 11 studies reported freestyle performance. Six studies reported VO_2_max, three reported HRmax, and three reported VEmax. In addition, in four studies, data were extracted from figures rather than directly reported (Campos et al., 2017; Swanwick and Matthews, 2018; McGuinness and Higgins, 2020; Deeks et al., 2025).

**FIGURE 2:**
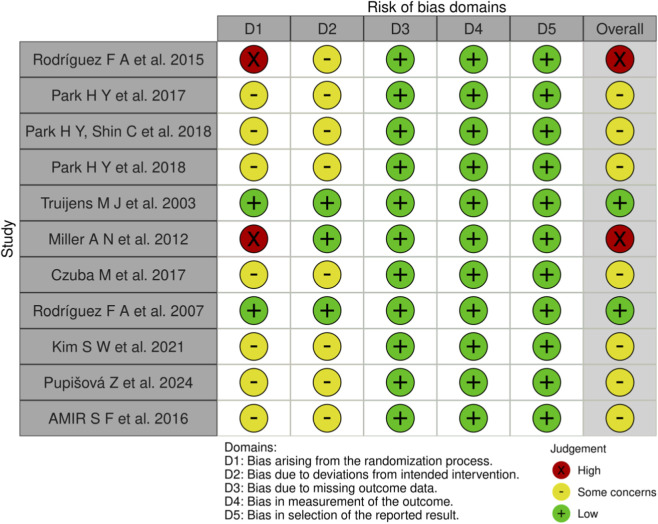
Summary of risk-of-bias judgments across individual studies using the RoB 2 tool.

**FIGURE 3:**
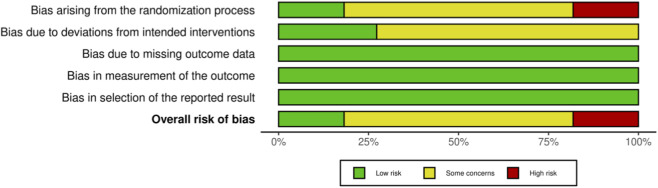
Proportion of included studies rated as low risk, some concerns, or high risk in each RoB 2 domain.

The RoB 2 tool was applied to assess the risk of bias in the 11 included studies, with the results presented in Figures 2, 3. Overall methodological quality across the 11 studies was rated as moderate. Specifically, two studies were judged to be at “low risk of bias,” seven at “some concerns” due to insufficient implementation of randomization and blinding, and two at “high risk of bias” owing to the absence of both random allocation and blinding. At the domain level, most studies were rated as low risk of bias for missing outcome data, outcome measurement, and selective reporting. However, concerns frequently arose in the domains of randomization and deviations from intended interventions. These findings highlight the need for future intervention studies to adopt more rigorous designs, including strict adherence to randomization procedures and the implementation of blinding.

### 3.2 Overall pooled analysis

**FIGURE 4:**
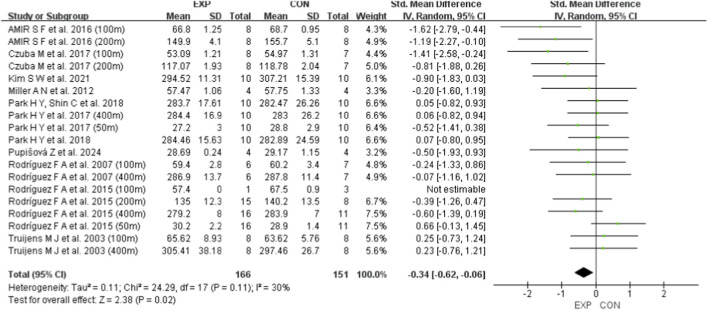
A meta-analysis of the effects of HI on swimming performance. Mean mean, SD standard deviation, Total sample size, Std. Mean Difference (SMD) effect size, CI confidence interval, df degrees of freedom, IV inverse variance, Random random pooling model.

**FIGURE 5:**
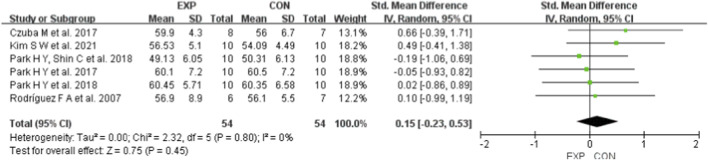
A meta-analysis of the effects of HI on VO_2_max. Mean mean, SD standard deviation, Total sample size, Std. Mean Difference (SMD) effect size, CI confidence interval, df degrees of freedom, IV inverse variance, Random random pooling model.

**FIGURE 6:**
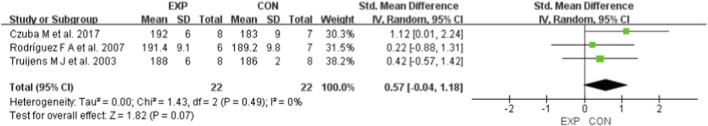
A meta-analysis of the effects of HI on HRmax. Mean mean, SD standard deviation, Total sample size, Std. Mean Difference (SMD) effect size, CI confidence interval, df degrees of freedom, IV inverse variance, Random random pooling model.

**FIGURE 7:**
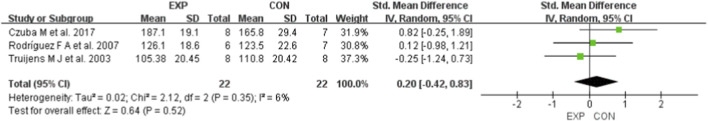
A meta-analysis of the effects of HI on VEmax. Mean mean, SD standard deviation, Total sample size, Std. Mean Difference (SMD) effect size, CI confidence interval, df degrees of freedom, IV inverse variance, Random random pooling model.

A total of 11 studies were included to evaluate the effects of HI on swimming performance, VO_2_max, HRmax, and VEmax in swimmers. Regardless of sex, age, or intervention characteristics, the pooled results showed that HI had a small but statistically significant effect on swimming performance, with an SMD of −0.34 (95% CI: [−0.62, −0.06], p = 0.02; χ^2^ = 24.29, p = 0.11; I^2^ = 30%) (Figure 4). In contrast, the effects on VO_2_max (SMD = 0.15; 95% CI: [−0.23, 0.53], p = 0.45; χ^2^ = 2.32, p = 0.80; I^2^ = 0%) (Figure 5), HRmax (SMD = 0.57; 95% CI: [−0.04, 1.18], p = 0.07; χ^2^ = 1.43, p = 0.49; I^2^ = 0%) (Figure 6), and VEmax (SMD = −0.20; 95% CI: [−0.42, 0.83], p = 0.52; χ^2^ = 2.12, p = 0.35; I^2^ = 6%) (Figure 7) were not statistically significant. These findings indicate that HI produced a small but significant effect on swimming performance (0.2 ≤ SMD <0.49), whereas no statistically significant effects were observed for VO_2_max, HRmax, or VEmax (all p > 0.05). HeterogSeneity across all outcomes was low, with I^2^ values below 40%.

To assess the robustness of the findings and potential publication bias, leave-one-out sensitivity analyses were performed, funnel plots were generated, and Egger’s test was conducted (Supplementary Appendix C). The results showed that no single study disproportionately influenced the overall estimates, suggesting that the observed effects of HI on swimming performance, VO_2_max, HRmax, and VEmax were not driven by any individual study. The conclusions remained stable after sequential exclusion of each study. Moreover, the funnel plots appeared symmetrical, and Egger’s test did not detect significant publication bias (all p > 0.05), indicating no substantial reporting bias among the included studies.

### 3.3 Subgroup analysis

Table 2 presents the subgroup effects of HI on swimming training outcomes. For swimming events, HI had the largest effect on the 400 m freestyle (MD = −4.205, p = 0.059), although it was not statistically significant, followed by the 200 m freestyle (MD = −3.101, p = 0.037). In terms of simulated altitude, elevations ≥3500 m showed the greatest effect on swimming performance (SMD = −0.719, p = 0.017).

**TABLE 2:** Subgroup analysis of the effects of HI. FR freestyle.

Subgroup	Category	Heterogeneity test		Effect size [95%CI]	Two-tailed test		Studies (swimming performance index) included
		I^2^	p		Z	P	
Swimming event	50 m FR	64.5%	0.060	−0.046 [-1.574, 1.482]	−0.059	0.953	3 (3)
	100 m FR	0.0%	0.413	−1.502 [-2.224, −0.780]	−4.078	0.000	5 (5)
	200 m FR	29.7%	0.241	−3.101 [-6.009, −0.193]	−2.090	0.037	3 (3)
	400 m FR	0.0%	0.707	−4.205 [-8.571, 0.161]	−1.888	0.059	7 (7)
Simulated high altitude	≤2500 m	44.3%	0.084	−0.238 [-0.700, 0.224]	−1.010	0.312	4 (8)
	3000 m	0.0%	0.452	−0.229 [-0.627, 0.169]	−1.129	0.259	4 (5)
	≥3500 m	22.5%	0.271	−0.719 [-1.309, −0.129]	−2.387	0.017	3 (5)

## 4 Discussion

The present systematic review and meta-analysis provides novel evidence regarding the effects of HI on swimming performance in competitive athletes. We found that HI exerted a small but statistically significant positive effect on swimming performance, whereas no significant changes were observed for VO_2_max, HRmax, or VEmax. These findings indicate that the beneficial impact of HI on swimming is evident at the level of competition performance but does not appear to be mediated by large improvements in conventional cardiopulmonary markers. However, the interpretation of these findings should be tempered by the methodological quality of the available evidence. A substantial proportion of included studies were rated as having “some concerns” or “high risk” of bias, primarily due to limitations in randomization procedures and potential deviations from intended interventions. These issues may introduce uncertainty regarding the precise magnitude of the observed effects, even though they are less likely to alter the overall direction of the performance outcomes. Additionally, multiple studies rely on data extracted from charts using WebPlotDigitizer, which may affect results when the overall sample size is small. Accordingly, the observed benefits of hypoxic interventions should be interpreted as supported by evidence of moderate certainty, with greater confidence in the consistency of the effect than in its exact size. It should be noted that the umbrella term “hypoxic interventions” in this meta-analysis encompasses several training paradigms with distinct emphases. Broadly, the included studies can be classified into three functional categories: (1) hypoxic exposure–dominant approaches, which primarily aim to induce systemic hypoxic stress (Millet et al., 2010; Wilber et al., 2007); (2) hypoxic training–integrated endurance or interval protocols, targeting peripheral metabolic adaptations and fatigue resistance (Czuba et al., 2017; Park et al., 2018a); and (3) high-intensity or repeated-sprint training in hypoxia, emphasizing neuromuscular and anaerobic stress to enhance speed endurance (Brocherie et al., 2017; Camacho-Cardeñosa et al., 2020). Although these paradigms differ in their underlying mechanisms and training objectives, they share the common principle of using reduced oxygen availability to amplify training stress beyond normoxic conditions.

One of the most notable patterns emerging from this synthesis was the divergence between significant improvements in race performance and the absence of corresponding changes in VO_2_max, HRmax, and VEmax. This discrepancy implies that HI in swimmers may primarily induce non-hematological adaptations rather than large-scale systemic changes in oxygen transport. Swimming is performed in a constrained breathing environment with higher energetic costs of propulsion compared to land-based endurance sports (Burtscher et al., 2022; Schmidt and Prommer, 2010). Under such conditions, HI is likely to promote adaptations at the muscular and neuromuscular level, including improved tolerance to metabolite accumulation, enhanced buffering capacity, greater muscle oxygen utilization, and refined pacing strategies (Tomažin et al., 2020). At the mechanistic level, first, VO_2_max is primarily determined by central oxygen transport capacity, including maximal cardiac output and total hemoglobin mass. Meaningful adaptations in these parameters typically require prolonged or sustained hypoxic exposure (Wilber et al., 2007; Schmidt and Prommer, 2010). In the present review, most interventions relied on intermittent or training cycle–based hypoxic stimuli with relatively short mesocycle durations (3–8 weeks), which may be insufficient to induce detectable increases in VO_2_max, particularly in well-trained swimmers who are already close to their physiological ceiling. Second, HRmax is relatively insensitive to training-induced adaptations and is more strongly influenced by age and autonomic regulation than by hypoxic exposure or endurance training *per se* (Millet et al., 2010; Buchheit and Laursen, 2013). Accordingly, the absence of changes in HRmax observed across studies is consistent with established principles of training physiology and does not preclude the presence of meaningful performance adaptations. Third, maximal ventilation reflects both pulmonary mechanics and central ventilatory drive. Unless hypoxic exposure is sustained or combined with targeted respiratory muscle loading, intermittent hypoxic training is unlikely to elicit substantial changes in either component (Álvarez-Herms et al., 2019). In swimming, where breathing patterns are constrained by stroke mechanics and race pacing, performance improvements may therefore occur even in the absence of concomitant increases in VEmax. The data therefore suggest that HI in swimming may act less as a vehicle for increasing aerobic capacity and more as a means of training the ability to sustain output and resist fatigue under oxygen-restricted conditions, which subsequently transfers to sea-level race environments (Just-Borràs et al., 2021).

Beyond conventional cardiopulmonary indices, several complementary physiological mechanisms may explain why hypoxic interventions can enhance swimming performance without eliciting significant changes in VO_2_max, HRmax, or VEmax. At the muscular level, hypoxic training has been shown to improve muscle oxygen utilization, thereby enhancing the capacity to sustain high-intensity exercise under metabolic stress (Brocherie et al., 2017; Bonetti and Hopkins, 2009). Repeated exposure to hypoxia may also augment buffering capacity and increase tolerance to metabolite accumulation, potentially delaying fatigue during speed-endurance events such as the 100–200 m swimming races. Concurrently, neuromuscular adaptations may further contribute to performance gains. Hypoxic and repeated-sprint training modalities have been demonstrated to enhance motor unit recruitment efficiency, modify neural firing patterns, and preserve force output under fatigued conditions, which may translate into more effective propulsion and better maintenance of stroke mechanics throughout a race (Millet and Brocherie, 2020; Tomažin et al., 2020). Notably, swimming performance is not determined solely by physiological capacity but is also influenced by task-specific and technical factors, including pacing strategies, stroke coordination, and the ability to maintain propulsive efficiency under constrained breathing conditions (Hellard et al., 2018; Peyrebrune et al., 2014). Hypoxic exposure may indirectly optimize these elements by increasing perceptual load and prompting swimmers to refine movement economy and race execution. Taken together, hypoxic interventions appear to exert their primary effects through the integration of peripheral neuromuscular and task-specific pathways rather than through large-scale central cardiopulmonary remodeling. This framework provides a plausible explanation for the dissociation observed in the present meta-analysis between improvements in competitive performance and the absence of concomitant changes in maximal physiological indices.

The subgroup analyses add further depth to this interpretation by highlighting the distance-dependent effects of HI. The largest and most consistent improvements were observed in 100 m and 200 m freestyle, where both statistical and practical significance were present. These events demand high levels of speed endurance, where the balance between anaerobic contribution and aerobic recovery is critical (Shoman, 2012). HI likely provides a competitive edge in these races by strengthening the ability to maintain speed despite limited recovery opportunities (Wądrzyk et al., 2022a). In contrast, 400 m freestyle showed a trend toward improvement, but statistical significance was not reached, which may be attributed to insufficient intervention duration or variability in training design across studies. The shorter 50 m sprint did not benefit from HI, a finding that is plausible given that such ultra-short events are dominated by technical execution in the start, underwater, and breakout phases (Wądrzyk et al., 2022b). Unless hypoxic protocols are explicitly designed to target these technical determinants, their contribution to total performance in the 50 m race is likely minimal (Avtandilyan et al., 2024). This distance-specific pattern underscores the importance of tailoring HI prescriptions to the unique physiological and technical requirements of each event.

Another key finding was the influence of simulated altitude on outcomes. Subgroup analysis revealed that protocols conducted at ≥3500 m demonstrated significant improvements in swimming performance, whereas interventions performed at ≤2500 m or 3000 m did not. This observation suggests the presence of a stimulus threshold: low to moderate altitudes may be insufficient to trigger adaptations that translate into measurable improvements at sea level, while higher hypoxic stress appears necessary to elicit beneficial effects (Robertson et al., 2010). From a physiological perspective, greater simulated altitudes induce a more pronounced reduction in arterial oxygen saturation, thereby intensifying metabolic stress during training and enhancing reliance on anaerobic pathways and peripheral oxygen extraction (Wilber et al., 2007; Bonetti and Hopkins, 2009). When combined with high-intensity interval or repeated-sprint training, such hypoxic stress may preferentially promote adaptations related to buffering capacity, fatigue tolerance, and the ability to sustain race pace under oxygen-restricted conditions, which are particularly relevant for speed-endurance swimming events (Millet and Brocherie, 2020; Brocherie et al., 2017). Nevertheless, higher simulated altitudes may also impose greater fatigue and recovery demands, emphasizing the need for careful monitoring and balance between stimulus and recovery (Ratz et al., 2009). Coaches should therefore consider both the magnitude of hypoxic stress and its integration into overall periodized training plans to maximize benefits while minimizing risks of overreaching.

In addition, variables such as age, sex, and training dose may moderate the effects of hypoxic interventions on swimming performance. Age represents a plausible moderator, as adaptive responses to hypoxic and high-intensity training vary across developmental stages, with younger athletes potentially exhibiting distinct neuromuscular and metabolic plasticity compared with adults (Millet and Brocherie, 2020; Wilber et al., 2007). However, most included studies involved late-adolescent or adult swimmers, with limited representation of younger cohorts, precluding age-specific analyses. Similarly, sex-related differences in ventilatory control, hematological responses, and fatigue tolerance under hypoxic conditions have been documented in endurance populations (Burtscher et al., 2022; Schmidt and Prommer, 2010). Yet, in the present synthesis, male participants predominated, and sex-stratified reporting specific to swimming trials was largely absent, preventing a formal assessment of sex as a moderating factor. Training frequency and intervention duration also warrant consideration, as hypoxic adaptations are known to depend on the cumulative “dose” of exposure and training stress (Wilber et al., 2007). Across the included studies, intervention periods ranged from 3 to 8 weeks, with substantial variability in weekly training frequency, which may partly explain differences in effect magnitude despite relatively low overall statistical heterogeneity. Shorter intervention durations or lower weekly training volumes may be insufficient to elicit adaptations that translate meaningfully into competitive performance, particularly in speed-endurance–dominant events. Owing to the limited number of studies and inconsistent reporting of training load and adherence, formal dose–response or meta-regression analyses were not feasible. Consequently, while the present findings support a beneficial effect of hypoxic interventions on swimming performance, the extent to which age, sex, training frequency, and intervention duration moderate these effects remains an important target for future, more rigorously designed trials.

From a practical perspective, the magnitude of effect observed in this review, although classified as small, should not be underestimated in competitive swimming, where race outcomes are often determined by tenths of a second. When interpreted in relation to the typical variability of race times reported in the included studies, the pooled standardized effects correspond approximately to improvements on the order of 1.5–3 s in the 100 m and 200 m freestyle events. These values should be regarded as indicative of potential practical relevance rather than precise or universally generalizable performance gains, particularly given the limited number of studies contributing to each event-specific subgroup. Moreover, the low heterogeneity observed across most outcomes suggests that the direction of effect is relatively consistent despite variability in intervention design, lending further confidence to the practical relevance of these findings. At the same time, the current evidence highlights the critical importance of intervention duration, training frequency, and the structuring of work-to-rest ratios. The majority of included studies used mesocycles of 3–8 weeks, with 3–18 sessions per week, and most sessions lasting at least 60 min. These conditions may represent a minimum threshold for inducing meaningful adaptations, particularly for middle-distance events such as the 400 m.

The overall methodological quality of included studies was moderate, with the main concerns relating to randomization procedures and deviations from intended interventions. This limitation emphasizes the need for future trials to adopt more rigorous designs, including proper random allocation, blinding of outcome assessors, and standardized reporting of adherence and adverse events. Despite these shortcomings, the consistency of the performance results and the stability of the effect sizes across sensitivity analyses provide reasonable confidence in the main conclusions. Still, it remains critical for future research to not only improve methodological rigor but also to systematically capture mechanistic variables such as muscle oxygenation dynamics, metabolic markers, and technical efficiency in order to better explain why HI is effective in swimmers.

## 5 Limitations

Several limitations of this review should be acknowledged. First, given the current sample size limitations, this may affect the statistical power and Type I error of subgroup analysis, and the results may not be applicable to non-elite swimmers. Second, All swimming performance metrics included in this study were measured in freestyle, so the aggregated results may not be applicable to other swimming strokes. Third, there was substantial variability in intervention characteristics, including simulated altitude, frequency, duration, and session structure, which complicates the identification of an optimal hypoxic “dose.” Although overall heterogeneity was low, the diversity of protocols still reduces the precision of conclusions regarding best practice. Fourth, methodological quality was rated as moderate, with shortcomings in randomization and blinding potentially introducing bias. Fifth, several studies required data extraction from graphs, which may have introduced measurement error. Finally, most interventions lasted only 3–8 weeks, which may not be sufficient to observe longer-term adaptations, particularly in events with a higher aerobic contribution such as the 400 m freestyle.

## 6 Conclusion

In conclusion, this systematic review and meta-analysis demonstrates that HI confer a small but significant improvement in swimming performance among competitive athletes, with the strongest evidence for benefits in the 100 m and 200 m freestyle events. In contrast, no significant effects were observed for VO_2_max, HRmax, or VEmax, suggesting that the mechanisms underlying these improvements are more likely related to muscular, metabolic, and technical adaptations rather than large-scale changes in oxygen transport capacity. Subgroup analyses indicate that higher simulated altitudes (≥3500 m) may represent a necessary threshold for eliciting beneficial adaptations, while shorter or lower-intensity interventions may be insufficient. These findings underscore the importance of tailoring hypoxic training to the specific demands of swimming events, integrating sufficient stimulus and recovery, and situating interventions within a broader periodized training framework. Future research should focus on defining the dose–response relationship of hypoxic training, extending interventions to longer durations, improving methodological rigor, and incorporating mechanistic assessments that capture the unique physiological and technical demands of swimming.

## Data Availability

The original contributions presented in the study are included in the article/Supplementary Material, further inquiries can be directed to the corresponding author.
