# Supplementary material for: The effect of hypoxic interventions on swimming performance in competitive athletes: a systematic review and meta-analysis
Source: Front Physiol. 2026 Feb 9;17:1755641. doi: 10.3389/fphys.2026.1755641 (PMC12926167; doi:10.3389/fphys.2026.1755641)
Supplement: Supplementary file 2 [file Supplementaryfile3.docx]

**Appendix C Correlation Chart**

**1 Publication Bias Assessment**


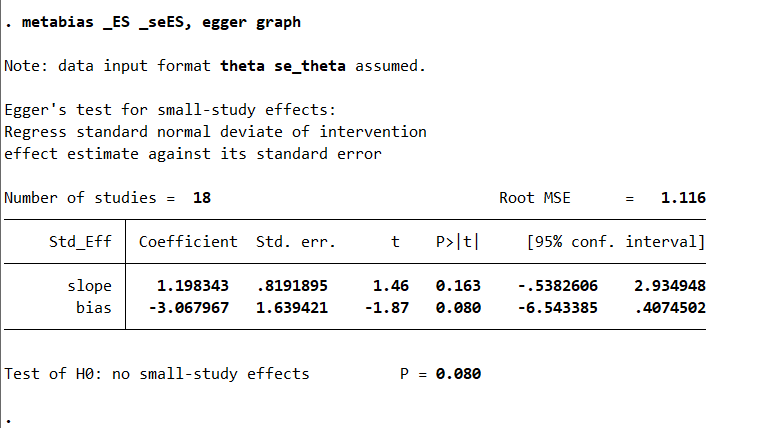


Egger’ test of swimming performance.


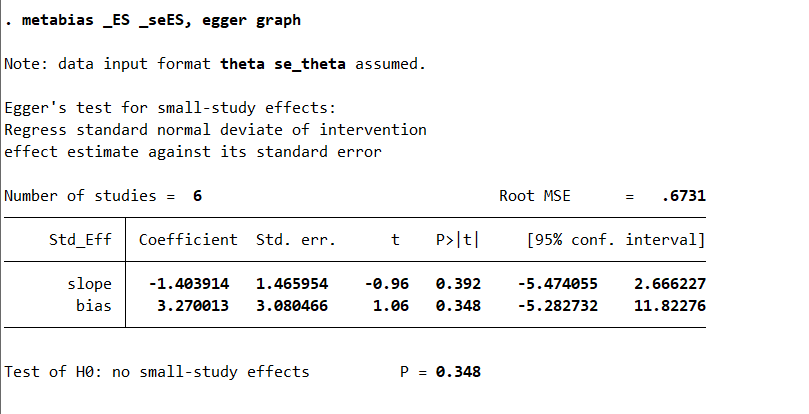


Egger’ test of VO2max.


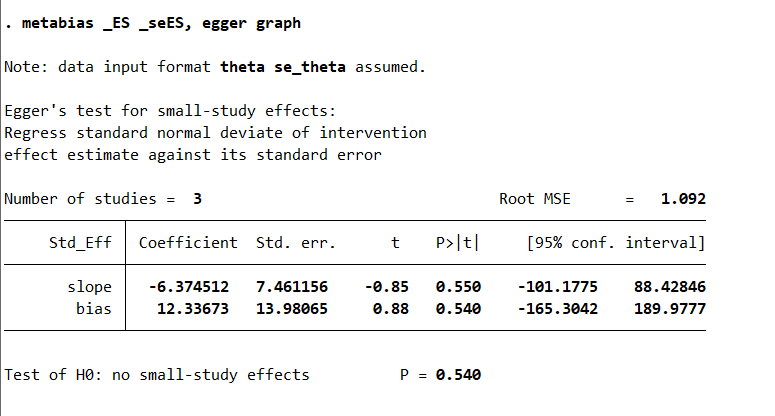


Egger’ test of VEmax.


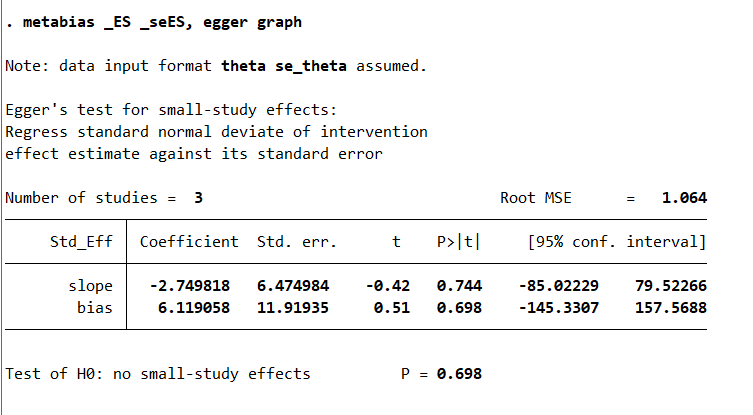


Egger’ test of HRmax.


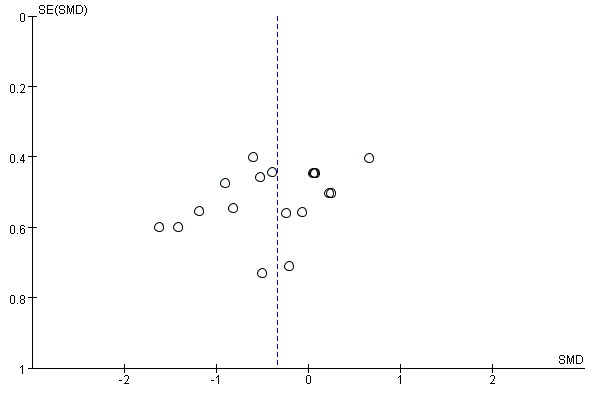


Funnel plot of swimming performance.


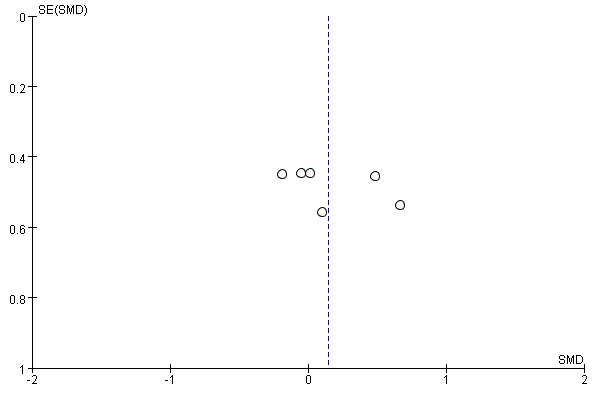


Funnel plot of VO2max.


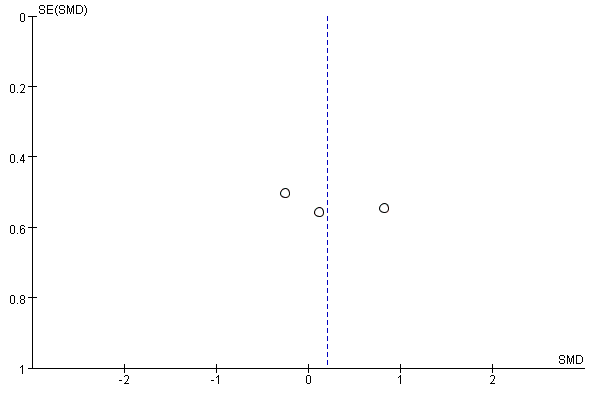


Funnel plot of VEmax.


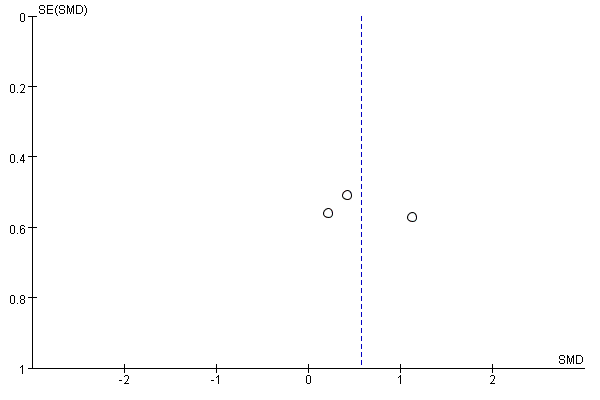


Funnel plot of HRmax.

**2 Sensitivity Analyses: Leave-One-Out for Key Outcomes**


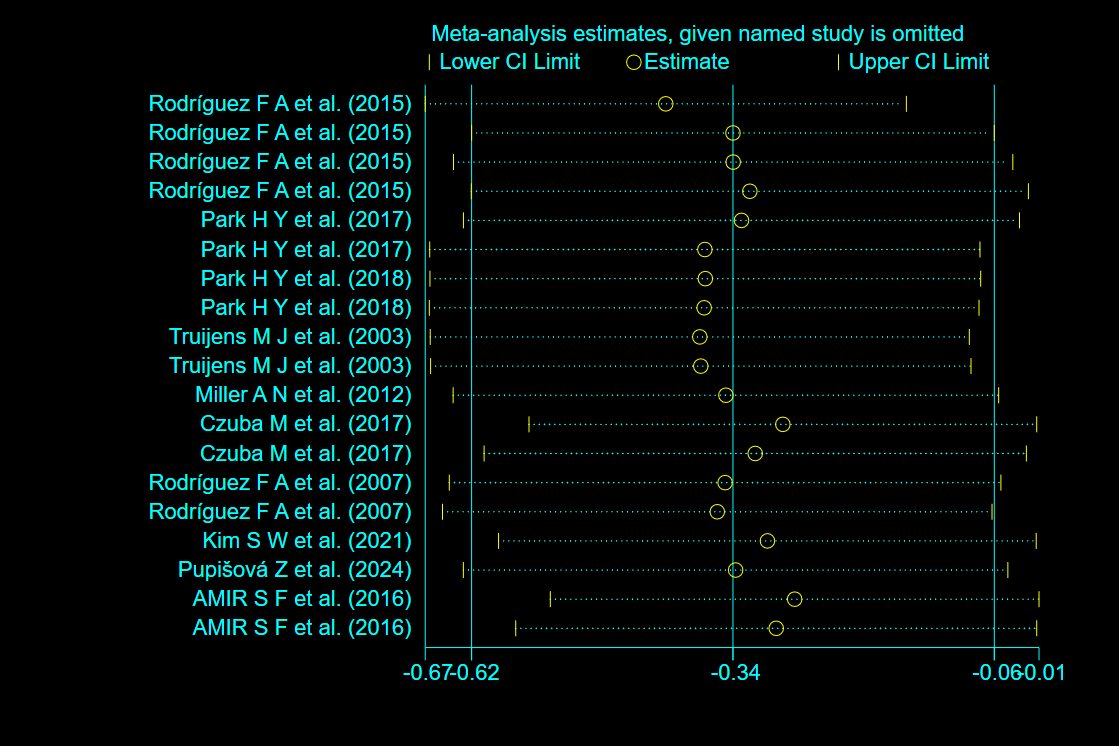


Leave-One-Out Sensitivity Analysis for swimming performance.


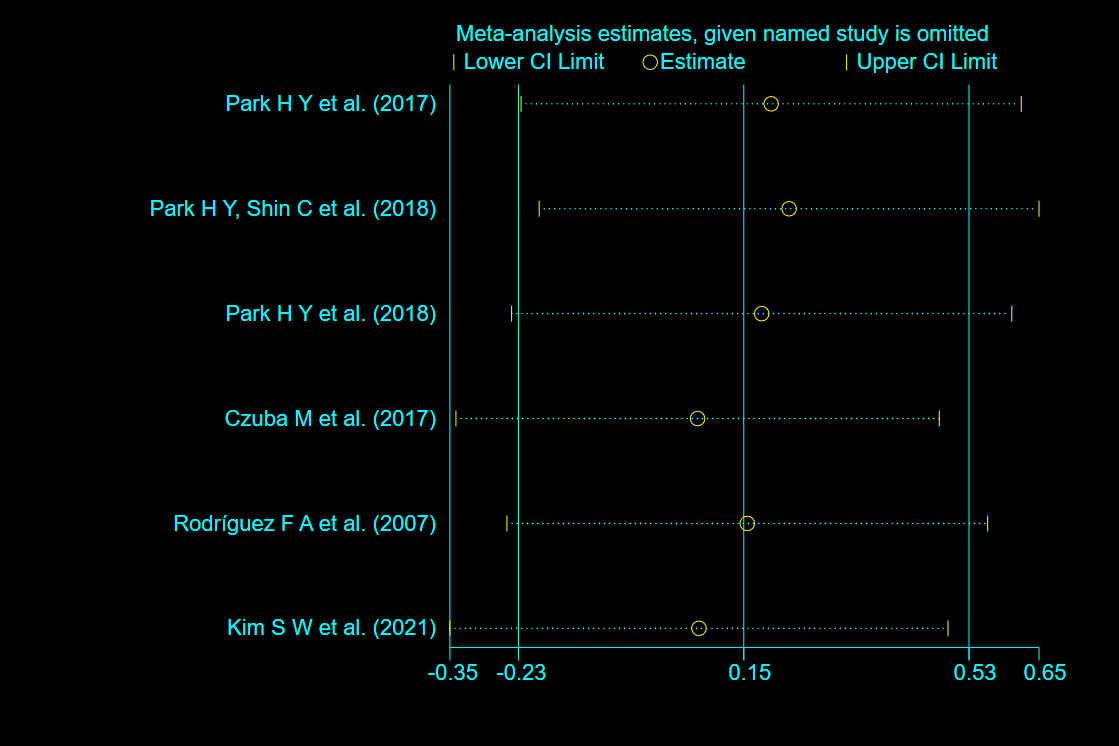


Leave-One-Out Sensitivity Analysis for VO2max.


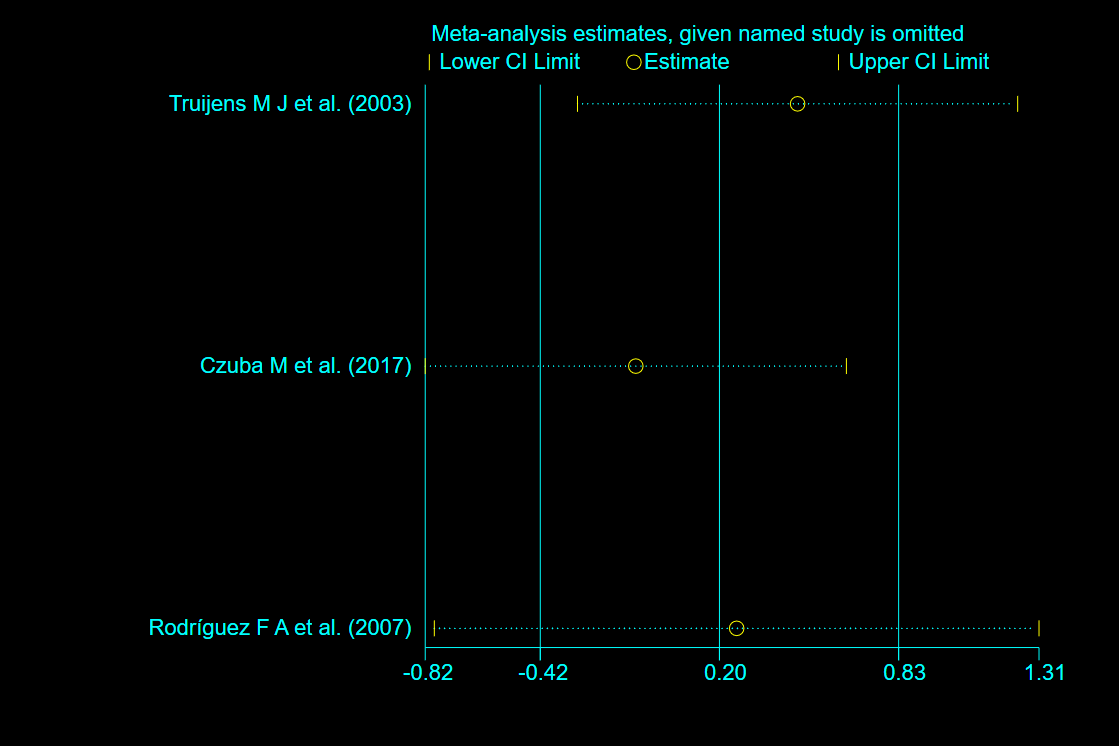


Leave-One-Out Sensitivity Analysis for VEmax.


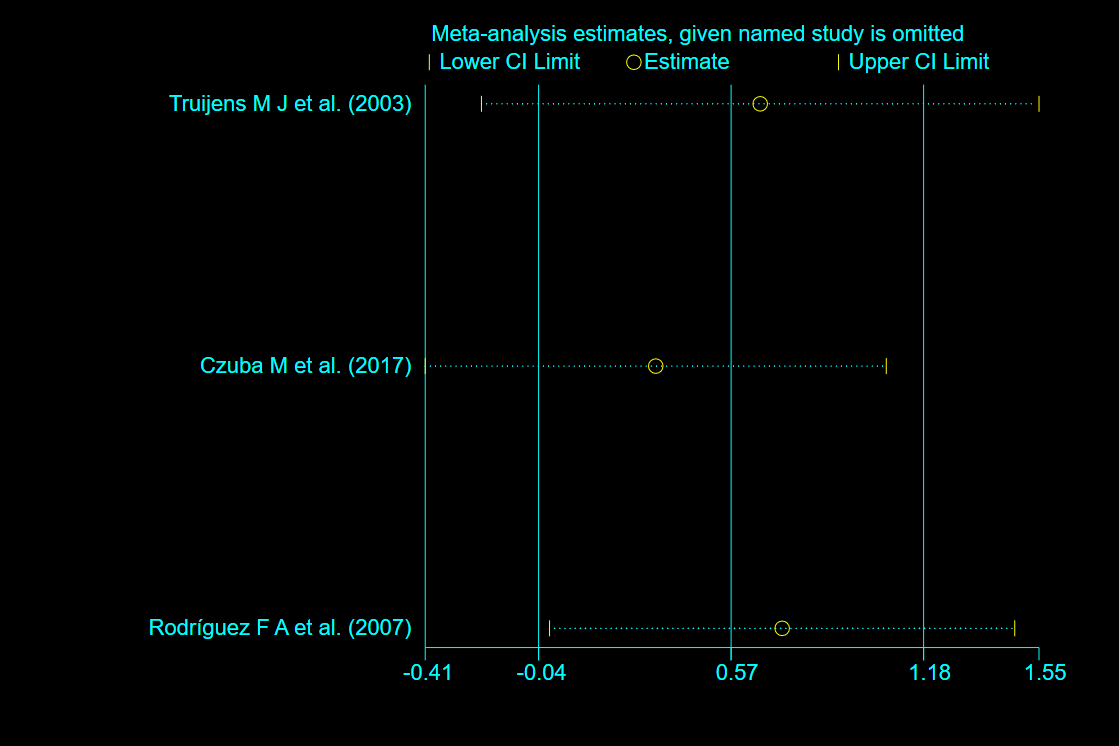


Leave-One-Out Sensitivity Analysis for HRmax.

**3 Subgroup analysis results**


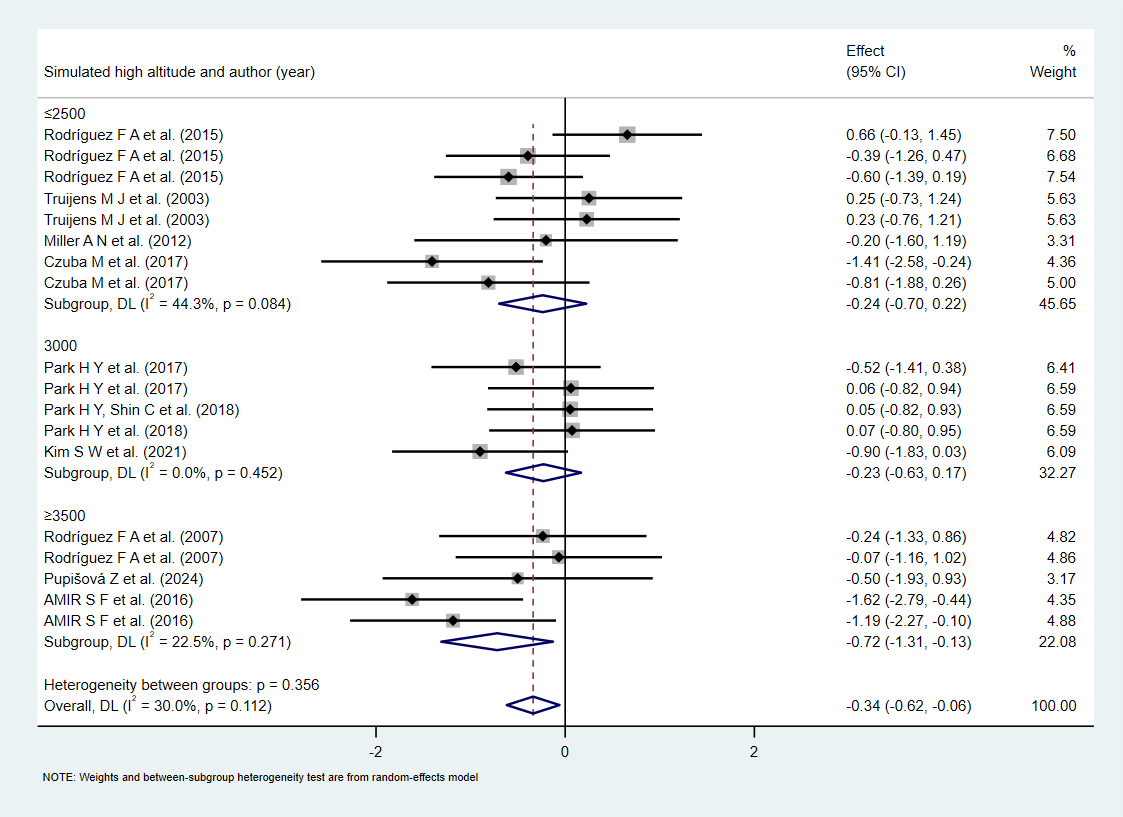


Subgroup results of the simulated height adjustment effect.


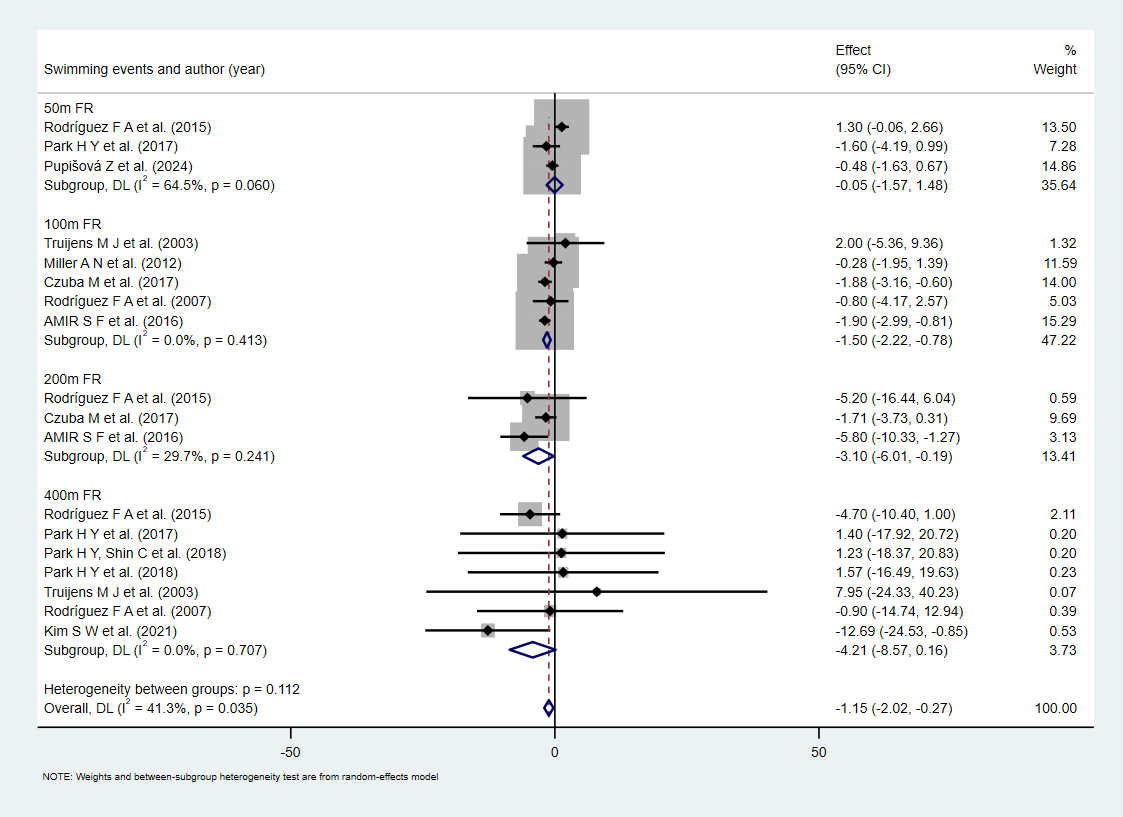


Subgroup results of the swimming event adjustment effect.

**4 Forest Map**

**
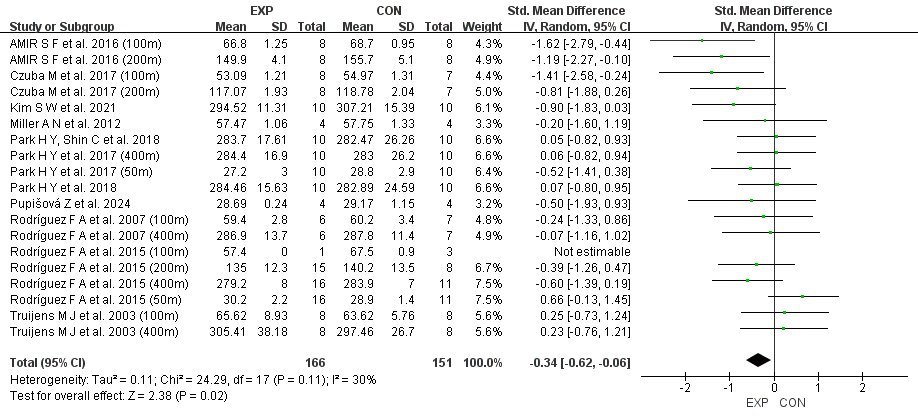
**

Forest Plot of Swimming Performance Metrics.

**
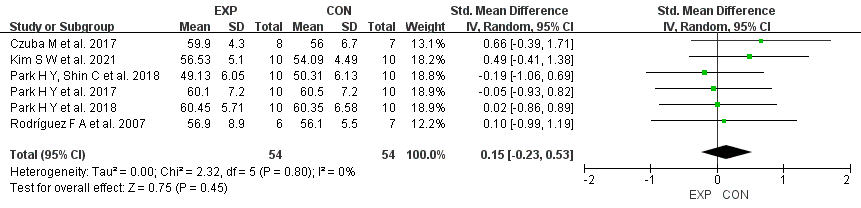
**

Forest Plot of VO_2_max Metrics.


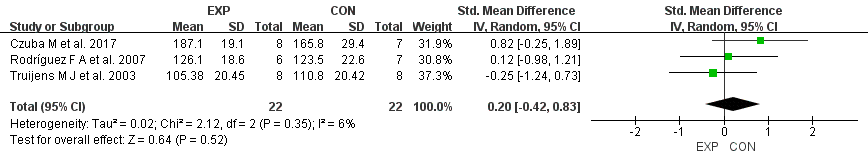


Forest Plot of VEmax Metrics.


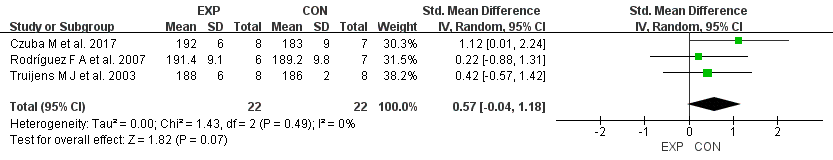


Forest Plot of HRmax Metrics.
