# Supplementary material for: The effect of hypoxic interventions on swimming performance in competitive athletes: a systematic review and meta-analysis
Source: Front Physiol. 2026 Feb 9;17:1755641. doi: 10.3389/fphys.2026.1755641 (PMC12926167; doi:10.3389/fphys.2026.1755641)
Supplement: Supplementary file 3 [file Supplementaryfile2.docx]

**Appendix B Database Search Terms**

**Web of Science**

TS = (hypoxi* OR "altitude training" OR "simulated altitude" OR "normobaric hypoxia" OR "hypobaric hypoxia" OR LHTL OR "live high train low") AND TS = (IHT OR "intermittent hypoxic training" OR "intermittent hypoxia training" OR HIIT OR "high-intensity interval" OR "high intensity interval" OR "sprint interval" OR sprint* OR "repeated sprint" OR RSH OR "exercise training") AND TS = (swim* OR swimmer* OR "swimming performance" OR breaststroke OR butterfly OR freestyle OR backstroke)

**ScienceDirect**

(hypoxi OR "altitude training" OR "live high train low" OR normobaric hypoxia) AND ("intermittent hypoxic training" OR "high intensity interval" OR "repeated sprint" OR "sprint interval") AND swimmer

**Proquest**

subject(hypoxi* OR "altitude training" OR "simulated altitude" OR "normobaric hypoxia" OR "hypobaric hypoxia" OR LHTL OR "live high train low") AND subject(IHT OR "intermittent hypoxic training" OR "intermittent hypoxia training" OR hint OR "high-intensity interval" OR "high intensity interval" OR "sprint interval" OR sprint* OR "repeated sprint" OR RSH OR "exercise training") AND subject(swim* OR swimmer* OR "swimming performance" OR breaststroke OR butterfly OR freestyle OR backstroke)

**PubMed**

((hypoxi* OR "altitude training" OR "simulated altitude" OR "normobaric hypoxia" OR "hypobaric hypoxia" OR LHTL OR "live high train low"[MeSH Major Topic]) AND (IHT OR "intermittent hypoxic training" OR "intermittent hypoxia training" OR HIIT OR "high-intensity interval" OR "high intensity interval" OR "sprint interval" OR sprint* OR "repeated sprint" OR RSH OR "exercise training"[MeSH Major Topic])) AND (swim* OR swimmer* OR "swimming performance" OR breaststroke OR butterfly OR freestyle OR backstroke[MeSH Major Topic])

**Embase**

(hypoxi* OR 'altitude training' OR 'simulated altitude' OR 'normobaric hypoxia' OR 'hypobaric hypoxia' OR lhtl OR 'live high train low') AND (iht OR 'intermittent hypoxic training' OR 'intermittent hypoxia training' OR hiit OR 'high-intensity interval' OR 'high intensity interval' OR 'sprint interval' OR sprint* OR 'repeated sprint' OR rsh OR 'exercise training') AND (swim* OR swimmer* OR 'swimming performance' OR breaststroke OR butterfly OR freestyle OR backstroke)

**Cochrane Library**

9 Trials matching hypoxi* OR "altitude training" OR "simulated altitude" OR "normobaric hypoxia" OR "hypobaric hypoxia" OR LHTL OR "live high train low" in Title Abstract Keyword AND IHT OR "intermittent hypoxic training" OR "intermittent hypoxia training" OR HIIT OR "high-intensity interval" OR "high intensity interval" OR "sprint interval" OR sprint* OR "repeated sprint" OR RSH OR "exercise training" in Title Abstract Keyword AND swim* OR swimmer* OR "swimming performance" OR breaststroke OR butterfly OR freestyle OR backstroke in Title Abstract Keyword - (Word variations have been searched)

[Cochrane Central Register of Controlled Trials](https://www.cochranelibrary.com/en/central/about-central)

**SPORTDiscus**

(hypoxi* OR "altitude training" OR "simulated altitude" OR "normobaric hypoxia" OR "hypobaric hypoxia" OR LHTL OR "live high train low") AND (IHT OR "intermittent hypoxic training" OR "intermittent hypoxia training" OR HIIT OR "high-intensity interval" OR "high intensity interval" OR "sprint interval" OR sprint* OR "repeated sprint" OR RSH OR "exercise training") AND (swim* OR swimmer* OR "swimming performance" OR breaststroke OR butterfly OR freestyle OR backstroke)
